# Green spaces and mortality: a systematic review and meta-analysis of cohort studies

**DOI:** 10.1016/S2542-5196(19)30215-3

**Published:** 2019-11

**Authors:** David Rojas-Rueda, Mark J Nieuwenhuijsen, Mireia Gascon, Daniela Perez-Leon, Pierpaolo Mudu

**Affiliations:** a Department of Environmental and Radiological Health Sciences, Colorado State University, Fort Collins, CO, USA; b ISGlobal, Centre for Research in Environmental Epidemiology, Barcelona, Spain; c Municipal Institute of Medical Research, Barcelona, Spain; d Universitat Pompeu Fabra, Barcelona, Spain; e CIBER Epidemiología y Salud Pública, Madrid, Spain; f Unidad Docente de Medicina Preventiva y Salud Pública, Hospital del Mar, Barcelona, Spain; g Department of Public Health, Environmental and Social Determinants of Health, World Health Organization, Geneva, Switzerland

## Abstract

**Background:**

Green spaces have been proposed to be a health determinant, improving health and wellbeing through different mechanisms. We aimed to systematically review the epidemiological evidence from longitudinal studies that have investigated green spaces and their association with all-cause mortality. We aimed to evaluate this evidence with a meta-analysis, to determine exposure-response functions for future quantitative health impact assessments.

**Methods:**

We did a systematic review and meta-analysis of cohort studies on green spaces and all-cause mortality. We searched for studies published and indexed in MEDLINE before Aug 20, 2019, which we complemented with an additional search of cited literature. We included studies if their design was longitudinal; the exposure of interest was measured green space; the endpoint of interest was all-cause mortality; they provided a risk estimate (ie, a hazard ratio [HR]) and the corresponding 95% CI for the association between green space exposure and all-cause mortality; and they used normalised difference vegetation index (NDVI) as their green space exposure definition. Two investigators (DR-R and DP-L) independently screened the full-text articles for inclusion. We used a random-effects model to obtain pooled HRs. This study is registered with PROSPERO, CRD42018090315.

**Findings:**

We identified 9298 studies in MEDLINE and 13 studies that were reported in the literature but not indexed in MEDLINE, of which 9234 (99%) studies were excluded after screening the titles and abstracts and 68 (88%) of 77 remaining studies were excluded after assessment of the full texts. We included nine (12%) studies in our quantitative evaluation, which comprised 8 324 652 individuals from seven countries. Seven (78%) of the nine studies found a significant inverse relationship between an increase in surrounding greenness per 0·1 NDVI in a buffer zone of 500 m or less and the risk of all-cause mortality, but two studies found no association. The pooled HR for all-cause mortality per increment of 0·1 NDVI within a buffer of 500 m or less of a participant's residence was 0·96 (95% CI 0·94–0·97; I^2^, 95%).

**Interpretation:**

We found evidence of an inverse association between surrounding greenness and all-cause mortality. Interventions to increase and manage green spaces should therefore be considered as a strategic public health intervention.

**Funding:**

World Health Organization.

## Introduction

The urban environment has been proposed to be a key health determinant worldwide.^1^ Half of the global population lives in urban environments, and changes to urban design, such as the introduction of green spaces, have been suggested to improve population health.^1^, ^2^, ^3^, ^4^, ^5^, ^6^, ^7^, ^8^ Green spaces are associated with more physical activity, physical or mental restoration and reduced stress, higher social capital, and ecosystem services, such as better air quality, less traffic noise, less heat-island effects, and more biodiversity.^6^, ^9^

Green spaces have also been associated with better mental health, and benefits to the immune system and to metabolism, improved pregnancy outcomes, a reduction in cardiovascular disease, and reduced premature mortality.^3^, ^5^, ^7^, ^10^, ^11^ However, green spaces are also linked to some health risks, such as exposure to allergens (such as pollen), pesticides, herbicides, vector-borne diseases transmitted by arthropods (such as Lyme disease or dengue), accidental injuries due to activities performed in green space areas, and excessive exposure to ultraviolet radiation.^6^, ^12^ But, overall, most of the epidemiological evidence indicates that exposure to green spaces could result in health benefits.^4^, ^5^, ^9^, ^13^

Most of the evidence of the health effects of green spaces has come from ecological and cross-sectional studies, but few data come from longitudinal studies.^4^, ^5^, ^8^, ^9^, ^13^ Different indices of green spaces have been used to assess exposures to these spaces in previous epidemiological studies,^6^ including perception of green spaces, the accessibility of green spaces, visits to green spaces, land use (as determined by satellite images) and so-called surrounding greenness (ie, the presence or absence of green space in close proximity to participants' residences) or the size of green space (as determined by land-cover maps), green space facilities, tree cover, and canopy density.^6^ The use of such different exposure definitions has made it difficult to quantitatively summarise the associations between green spaces and health.^5^, ^14^ However, in the past 10 years, several longitudinal studies^4^ have included similar exposure indices that have used satellite images. Stakeholders, such as policy makers, urban planners, and non-governmental organisations, have also become interested in green spaces as a healthy urban design element, but the absence of quantitative evidence on the health impacts of green spaces have hindered their implementation. Approaches such as health impact assessments have been used by policy makers and decision makers to successfully apply health evidence to policy making, and the provision of robust quantitative evidence from a meta-analysis regarding green space and health outcomes could facilitate the use of health impact assessment.

**Research in context**

**Evidence before this study**

We searched MEDLINE via PubMed for longitudinal studies published before Aug 20, 2019, with search terms related to green spaces (“green space”, “greenspace”, “greenness”, “greenery”, “wilderness”, “wild land”, “natural land”, “natural environment”, “municipal land”, “community land”, “public land”, “open land”, “wild space”, “municipal space”, “natural space”, “open space”, “municipal park”, “park”, “botanic park”, “park access”, “urban park”, “city park”, “park availability”, “public garden”, “natural neighbourhood”, “natural facilities”, “vegetation natural”, “belt green”, “wild area”, “trail green”, “natural area”, “green area”, “built environment”, “urban design”, “recreation resource”, “woodland”, “forest”, “shinrin-yoku”, “forest bathing”, “NDVI”, and “normalized difference vegetation”) combined with keywords related to mortality (“mortality” and “all-cause mortality”) and specific study types (“longitudinal studies” and “cohort studies”). We also extended the search to papers and reports cited in the literature but not in MEDLINE. The search was restricted to work published in English. We also contacted study authors to gather unpublished data. We included studies if their design was longitudinal; the exposure of interest was measured green space; the endpoint of interest was all-cause mortality; they provided a risk estimate (ie, a hazard ratio) and the corresponding 95% CI for the association between green space exposure and all-cause mortality; and they used normalised difference vegetation index (NDVI) as the exposure index for green spaces. Over the past 10 years, evidence has increasingly suggested that green spaces could have a protective effect on mortality.

**Added value of this study**

Previous meta-analyses have reported pooled effects of green spaces and mortality. However, to our knowledge, our systematic review and meta-analysis is the first and the most comprehensive synthesis to date on green spaces and all-cause mortality, and it is the first to focus specifically on cohort studies. Our meta-analysis also provides an exposure-response function between surrounding greenness and all-cause mortality per exposure unit of NDVI. With data from nine cohorts, comprising more than 8 million individuals from seven different countries (Australia, Canada, China, Italy, Spain, Switzerland, and the USA), we found an inverse association between exposure to surrounding greenness and all-cause mortality.

**Implications of all the available evidence**

The results of our meta-analysis support interventions and policies to increase green spaces as an approach to improve public health. Our analysis also provides an exposure-response function that can be used in future quantitative health impact assessments that aim to estimate all-cause mortality associated with policy scenarios that affect green spaces.

To our knowledge, only two meta-analyses^4^, ^8^ on green spaces and mortality have previously been published, which combined evidence from cross-sectional and cohort studies on green spaces and mortality. However, combining different study designs is not always appropriate, and cross-sectional studies have well documented limitations. Therefore, in our study, we focused on longitudinal studies, which provide more robust epidemiological evidence than cross-sectional, prevalence studies. Our meta-analysis of longitudinal studies has only become possible in the past few years because several such studies have been published that have used similar exposure indices (namely, the normalised difference vegetation index [NDVI]). The main aim of our meta-analysis was to obtain an exposure-response function between green spaces and all-cause mortality, from cohort studies, for a new green space health impact assessment approach for WHO. A requirement of the exposure-response function is that it is based on an easily obtainable green space index and on the best available epidemiological evidence.

## Methods

### Study design

In this systematic review and meta-analysis, we aimed to collate evidence on green spaces and mortality from longitudinal epidemiological studies.^15^ Specifically, we aimed to assess the impact of residential green spaces on all-cause mortality, but we excluded studies without a longitudinal assessment. Our meta-analysis focused on studies that used the NDVI as an exposure index for green spaces, since this index was the most common exposure definition used by longitudinal studies, based on a rough assessment of the literature, and it was the easiest index to obtain because these are open-source satellite data that are available for any geographical location. NDVI is a good indicator for the density of plant growth on Earth. An NDVI score is obtained by remote sensing and it is estimated by calculating the near-infrared radiation minus the visible radiation, divided by near-infrared radiation plus visible radiation. Calculations of NDVI for a given pixel of a satellite image always result in a number that ranges from −1 to +1; however, absence of green leaves gives a value close to 0. A 0 means no vegetation, scores close to +1 (ie, 0·8–0·9) indicate the highest possible density of green leaves, and scores close to −1 indicate water.

### Search strategy and selection criteria

We searched MEDLINE via PubMed using a combination of medical subject headings and free-text terms for conditions of interest. We searched for studies published before Aug 20, 2019, with search terms related to green spaces (“green space”, “greenspace”, “greenness”, “greenery”, “wilderness”, “wild land”, “natural land”, “natural environment”, “municipal land”, “community land”, “public land”, “open land”, “wild space”, “municipal space”, “natural space”, “open space”, “municipal park”, “park”, “botanic park”, “park access”, “urban park”, “city park”, “park availability”, “public garden”, “natural neighbourhood”, “natural facilities”, “vegetation natural”, “belt green”, “wild area”, “trail green”, “natural area”, “green area”, “built environment”, “urban design”, “recreation resource”, “woodland”, “forest”, “shinrin-yoku”, “forest bathing”, “NDVI”, and “normalized difference vegetation”) combined with keywords related to mortality (“mortality” and “all-cause mortality”) and specific study types (“longitudinal studies” and “cohort studies”; appendix p 4). We also extended the search to papers and reports cited in the literature but not in MEDLINE. The search was restricted to work published in English and studies in humans. We also manually cross-checked the results of the title and abstract searches, to remove duplicates.

Two investigators (DR-R and DP-L) independently screened the titles and abstracts, before coming to a consensus opinion, to determine whether studies should be included. Eligibility criteria were also applied to the full-text articles during the final selection. We included studies if their design was longitudinal; the exposure of interest was measured green space; the endpoint of interest was all-cause mortality; they provided a risk estimate (ie, a hazard ratio [HR]) and the corresponding 95% CI for the association between green space exposure and all-cause mortality; and they used NDVI as the exposure index for green spaces. If several published reports were from the same study, we included only the one with the most detailed information. When discrepancies occurred (in two instances), we reached an agreement between ourselves to make a final decision. We extracted the first author name, publication year, country, size of the cohort, population demographic characteristics, length of follow-up, number of deaths, green space exposure definition, confounders used in the models, outcome details, and adjusted HR with 95% CIs from each study. We also contacted study authors to gather unpublished data. Two investigators (DR-R and DP-L) confirmed all data entries and checked data from each study at least twice for completeness and accuracy.

### Quality assessment

We evaluated the risk of bias by means of a checklist developed by WHO^16^ and van Kempen and colleagues.^17^ We aimed to evaluate the risk of bias associated with exposure assessment, confounding, selection of participants, and health outcome assessment. For each study, two investigators (DR-R and DP-L) independently evaluated the risk of bias before coming to a consensus opinion. How we scored the studies on these items is shown in the appendix (p 5). From these scores, we calculated a risk of bias score. Studies that the two investigators gave different risk of bias scores to were discussed, to reach consensus on their scores. Publication bias was assessed with a funnel plot and trim-and-fill method (appendix pp 7–8).

### Outcomes

Our primary endpoint was the risk of all-cause mortality per increment of surrounding greenness of 0·1 NDVI in a buffer zone of 500 m or less from a participant's residence.

### Statistical analysis

HRs were used to measure the association of interest. From each study, we extracted an HR exposure-response function per NDVI unit of change. Exposure-response functions from all the studies were estimated assuming linearity and providing final HR pool estimates per 0·1 NDVI increment. Our meta-analysis pooled fully adjusted HRs reported from the studies with a residential surrounding buffer equal to or less than 500 m. For those studies that presented cumulative and contemporaneous HRs, the cumulative HRs were chosen to be pooled with other studies. The study-specific estimates were pooled using a random-effects model, under an assumption that all the studies included in the analysis are a random sample of all possible studies that meet the inclusion criteria for the review.^18^ Between-study heterogeneity was assessed with the *I*^2^ index, which describes the inconsistency of findings across studies in the meta-analysis and reflects the extent to which CIs from the different studies overlap with each other.^19^

Analyses were done with RStudio statistical software, version 1.0.143, 2016. This study is registered with PROSPERO, CRD42018090315.

### Role of the funding source

The funder of the study participated in selection of the study design and requested inclusion of a quality assessment analysis. The funder of the study had no role in data collection, data analysis, data interpretation, or writing of the report. The corresponding author had full access to all the data in the study and had final responsibility for the decision to submit for publication.

## Results

**Figure 1:**
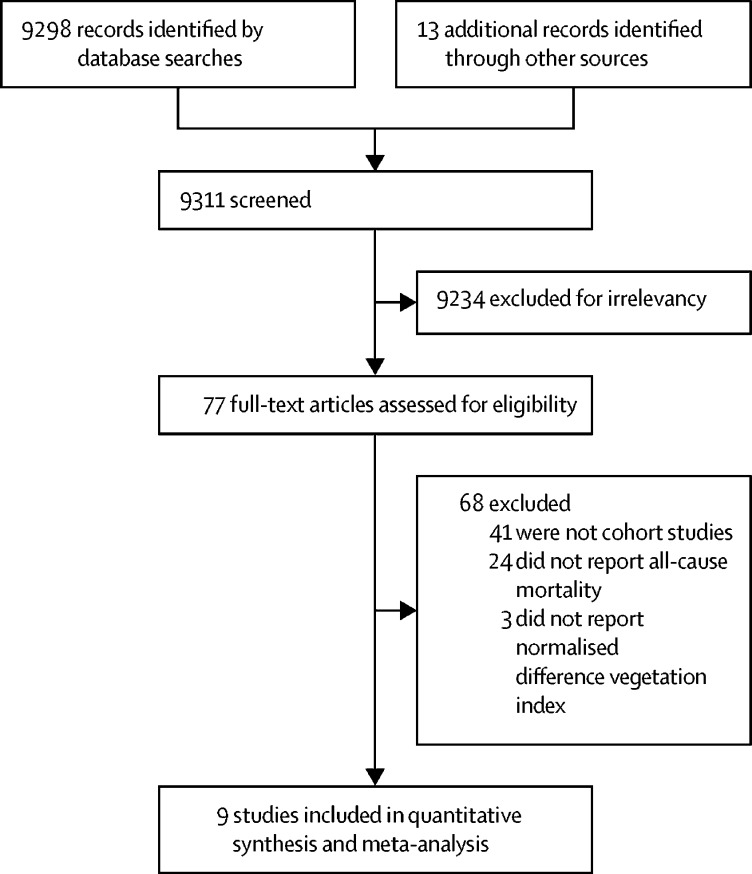
Study selection

We identified 9298 studies in MEDLINE and 13 studies that were reported in the literature but not indexed in MEDLINE (figure 1). We excluded 9234 (99%) studies after screening the titles and abstracts for duplications and for not meeting our inclusion criteria. After we reviewed the full texts of the remaining 77 (1%) studies, 41 (53%) studies were excluded because they were not cohort studies,^20^, ^21^, ^22^, ^23^, ^24^, ^25^, ^26^, ^27^, ^28^, ^29^, ^30^, ^31^, ^32^, ^33^, ^34^, ^35^, ^36^, ^37^, ^38^, ^39^, ^40^, ^41^, ^42^, ^43^, ^44^, ^45^, ^46^, ^47^, ^48^, ^49^, ^50^, ^51^, ^52^, ^53^, ^54^, ^55^, ^56^, ^57^, ^58^, ^59^, ^60^, ^61^ 24 (31%) were excluded because all-cause mortality was not reported,^7^, ^62^, ^63^, ^64^, ^65^, ^66^, ^67^, ^68^, ^69^, ^70^, ^71^, ^72^, ^73^, ^74^, ^75^, ^76^, ^77^, ^78^, ^79^, ^80^, ^81^, ^82^, ^83^, ^84^ and three (4%) were excluded because NDVI was not used as the exposure index.^85^, ^86^, ^87^ We included nine (12%) studies^88^, ^89^, ^90^, ^91^, ^92^, ^93^, ^94^, ^95^, ^96^ in our quantitative evaluation.

The nine studies in the quantitative evaluation were published between 2012 and 2019 (table). Of these studies, two were done in Canada,^88^, ^89^ two in the USA,^90^, ^91^ and the other five were done in Switzerland,^92^ China,^93^ Spain,^94^ Australia,^95^ and Italy.^96^ The sizes of the cohorts ranged from 1645 to 4 284 680 people (totalling 8 324 652 individuals). All cohort studies were done in adults (with age 21 years being the youngest age considered); and seven studies included both sexes, one only included women,^90^ and another only included men.^95^

**Table:** Studies included in the meta-analysis

	**Country**	**Study population**	Exposure definition*	**Exposure unit reported, normalised difference vegetation index score**	**Covariates adjusted for**	**Mortality outcome assessed**	**Deaths (%)**	**Hazard ratio (95% CI) per the exposure description**
Crouse et al (2017)^88^	Canada	1 265 515 (25–89 years, both sexes) for 10 years follow-up	250 m	IQR 0·15	Age, sex, ethnicity, marital status, education, income, employment, population density, particulate matter (PM_2·5_), ozone, and nitrogen dioxide	All non-accidental	106 180 (8·4%)	0·92 (0·91–0·93)
Villeneuve et al (2012)^89^	Canada	574 840 (>35 years, both sexes) for 22 years follow-up	500 m	IQR 0·24	Age, sex, city, household income, marital status, area measures of income, immigration and unemployment, distance to major roads and highways, nitrogen dioxide, and particulate matter (PM_2·5_)	All non-accidental	181 110 (31·5%)	0·95 (0·94–0·97)
James et al (2016)^90^	USA	108 630 (30–55 years, women) for 8 years follow-up	250 m	Per 0·1	Age, calendar year, ethnicity, marital status, parental occupation, husband's highest education, census-tract median home value and income, and smoking	All non-accidental	8604 (7·9%)	0·88 (0·82–0·94)
Wilker et al (2014)^91^	USA	1645 (>21 years, both sexes) for 5 years follow-up	250 m	IQR 0·22	Age, sex, race, smoking, coronary artery disease, stroke, atrial fibrillation, heart failure, diabetes, dyslipidaemia, hypertension, education, household income, and road distance to home	All-cause after stroke	929 (56·5%)	0·92 (0·81–1·05)
Vienneau et al (2017)^92^	Switzerland	4 284 680 (30–106 years, both sexes) for 8 years follow-up	500 m	IQR 0·14	Age, sex, marital status, job position, educational attainment, neighbourhood socioeconomic position, region, area type, altitude, particulate matter (PM_10_), and transport noise	Natural cause mortality	363 553 (8·5%)	0·94 (0·93–0·95)
Ji et al (2019)^93^	China	23 754 (≥80 years, both sexes) for 14 years follow-up	250 m	Per 0·1	Age, sex, ethnicity, marital status, geographical region, childhood socioeconomic status, adult socioeconomic status, social and leisure activity, smoking, alcohol consumption, and physical activity	All-cause mortality	18 948 (79·8%)	0·95 (0·94–0·95)
Nieuwenhuijsen et al (2018)^94^	Spain	792 649 (>18 years, both sexes) for 4 years follow-up	300 m	Per 0·1	Age, gender, socioeconomic status, and smoking	All-cause mortality	28 391 (3·6%)	0·92 (0·89–0·97)
Zijlema et al (2019)^95^	Australia	9218 (>65 years, men) for 18 years follow-up	300 m	Quartiles	Age, marital status, country of birth, education level, area-level socioeconomic status, and smoking	All-cause mortality	5889 (63·9%)	0·97 (0·89–1·05)
Orioli et al (2019)^96^	Italy	1 263 721 (>30 years, both sexes) for 12 years follow-up	300 m	Per 0·1	Age, sex, marital status, place of birth, education, occupation, and area-level socioeconomic position	All non-accidental	198 704 (15·7%)	0·99 (0·98–0·99)

* The zone of residential proximity to surrounding green space that was considered an exposure.

**Figure 2:**
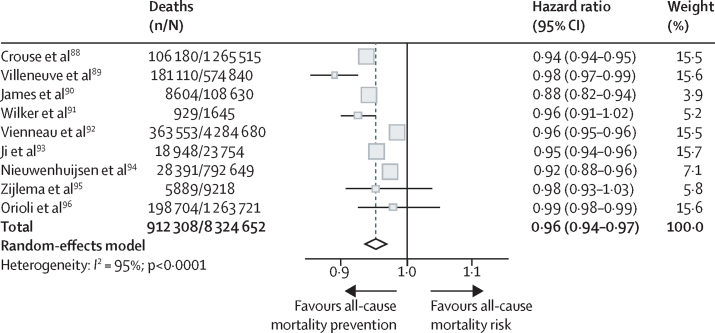
Meta-analysis of the association between greenness and all-cause mortality for each 0·1 increment of normalised difference vegetation index in less than 500 m from the participant's residence The size of the square for each estimated hazard ratio in the plot is proportional to the weight of the study, which indicates its relative impact on the calculations of the common effect. Some 95% CI lines are not visible because the data have narrow CIs.

Seven (78%) of the nine studies^85^, ^89^, ^90^, ^92^, ^93^, ^94^, ^96^ found a significant inverse relationship between an increase in surrounding greenness per 0·1 NDVI in a buffer zone of 500 m or less and the risk of all-cause mortality, but two studies^91^, ^95^ found no association (figure 2). Overall, the pooled HR of all-cause mortality was 0·96 (95% CI 0·94–0·97) for each increment of 0·1 NDVI in a residential buffer zone of 500 m or less (I^2^, 95%). Three studies^90^, ^92^, ^94^ were considered to have a high risk of bias (appendix p 6).

## Discussion

We found that increasing increments of residential greenness is significantly associated with reducing all-cause mortality in longitudinal studies. We included nine cohort studies in our quantitative evaluation, six of which^88^, ^89^, ^91^, ^93^, ^95^, ^96^ had a low risk of bias. This evidence supports interventions and policies to increase green spaces as an approach to improve public health.

The results of our study are consistent with previous systematic reviews and meta-analyses^4^, ^5^, ^6^, ^8^, ^97^ that had indicated a marked negative association between the amount of nearby green space and mortality and morbidity. Our study focused only on those epidemiological studies with longitudinal study design, providing more robust evidence and quantitative estimates for greenness that can be used for health impact assessment and risk assessment.

All the studies that we included were done in adults, with a study population of 1645–4 284 680 people (totalling 8 324 652 individuals across the studies) and a follow-up of 4–22 years. One study^90^ included only women and one study^95^ only included men, but the remainder involved people of both sexes. The study consisting of only women^90^ was given the lowest weighting in our analysis because of the presence of wider CIs in the study's HR. Finally, only one study^93^ in our meta-analysis was not done in a high-income country (China). In all the included studies, socioeconomic status (SES) was considered as a covariate. SES is important because of the possible variability in exposure to green space between different SES groups, since it is suggested that higher SES groups have more access to green spaces than lower SES groups.^98^ Although the HRs included in our analysis had been fully adjusted for covariates (including SES), we acknowledge that the variability in exposure to green spaces between different SES groups could still affect the results, and we cannot exclude fully residual confounding. All the included studies used NDVI as the exposure index, using buffer zones of 250–1250 m. We found an HR of 0·96 (95% CI 0·94–0·97) for all-cause mortality related to each 0·1 incremental unit of NDVI, indicating a protective effect of green spaces on mortality.

Several mechanisms, including biological pathways and health determinants, have been suggested as factors that might explain the health benefits conferred by green spaces.^99^, ^100^ However, establishing a causal relationship is difficult because the association between green spaces and health is complex.^101^, ^102^ Physical activity has been suggested as an important health determinant associated with green spaces.^5^, ^6^ Green space could be a place where physical activity for leisure can occur. Green spaces can also increase active transportation (walking and cycling).^1^ However, one of the studies^93^ that we included in our meta-analysis did a mediation analysis, which found that physical activity explained only 2% of the association between green spaces and mortality. Therefore, there must be other, more powerful mechanisms explaining our results. Through ecosystem services, green spaces can also confer several health effects. Attenuation of air pollution, noise, and heat-island effects are pathways that have been related to the protective effect of green spaces.^103^ Trees and other vegetation can decrease concentrations of air pollutants, and they can reduce atmospheric carbon dioxide through carbon storage and sequestration.^104^, ^105^ Three studies^88^, ^89^, ^92^ included air pollution as a covariate, although the HR of these studies did not differ substantially from studies that did not consider air pollution as a covariate. James and colleagues^90^ included air pollution in their mediation analysis, finding that PM_2·5_ could explain 4% of the association between green space and mortality. In a study in India, Pathak and colleagues^106^ showed that vegetation belts reduce traffic noise, a factor that has been related to cardiovascular health outcomes, stress, sleep disturbance, and mortality.^107^ Only one study^92^ that we included considered transport noise as a covariate. Green spaces have also been linked with an average cooling effect of 1°C in urban areas,^108^ which could partly explain the benefits of green spaces on mortality; however, notably, none of the included studies considered heat-island effects.

Stress reduction and improved relaxation and restoration are also pathways that have been suggested to explain the health benefits of green spaces.^6^ The psychosomatic stress reduction theory is one explanation of the benefits observed from green space exposure. This theory proposes that contact with nature (such as views of natural settings) can have a positive effect among those with high levels of stress by shifting them to a more positive emotional state.^6^, ^97^, ^109^ In a 2016 cross-sectional study^25^ in Barcelona (Spain), the relationship between subjective general health and greenness exposure was mediated, in part, by mental health status and enhanced social support. This study also found that this mediation effect could vary by sex and age. Another study^110^ performed in four Dutch cities found that stress and social cohesion were the strongest mediators between urban greenery and perceived general health. Finally, immune function has also been related to green spaces.^111^ Li and colleagues^48^, ^49^ found an association between visiting forests and improvement in the immune responses, including expression of anticancer proteins (such as perforin, granulysin, and granzymes A and B). An immunoregulation pathway through exposure to diverse microorganisms in the natural environments has also been proposed.^112^

Our systematic review and meta-analysis was restricted by the low availability and quality of published evidence on longitudinal studies between green space and mortality. Most of the evidence published on green spaces are from cross-sectional or ecological studies. We also had to exclude three studies because the exposure assessment definition was different from the other cohorts, and the estimates could not be combined. In our study, we assessed the publication bias through funnel plot and trim-and-fill methods (appendix pp 7–9). From both methods, we found low asymmetry, with almost no effect to the trim and fill estimations (HR 0·96, 95% CI 0·94–0·97). NDVI was used as the main exposure index for green spaces in our study. Although satellite-based measures of vegetation have been used extensively to measure exposure to greenness, NDVI does not measure the quality of greenness or accessibility to such green spaces, which are notable limitations. Heterogeneity between the studies is another limitation in our meta-analysis, since the studies included have different populations, such as sex-specific cohorts,^90^, ^95^ different age groups,^93^ and different buffer sizes. This heterogeneity should be taken into account when interpreting the pooled risk estimates.^113^, ^114^

As mentioned, many pathways have been proposed to explain the health effects of green spaces. Unfortunately, only one study^90^ included a mediation analysis, and all the other studies did not include an assessment of the proportion of the association explained by other health determinants, such as physical activity, air pollution, noise, and social capital. One assumption included in our meta-analysis was the linearity of the exposure-response function. Although all the cohort studies included in the meta-analysis provided an HR for continuous green space exposure, it is unlikely that the exposure-response function is linear, so further studies should test for non-linearity. Another limitation of our study was our restriction to studies published in English, creating a geographical bias, since most studies were from north America or western Europe. All the studies included also focused only on long-term residential exposure to green spaces and mortality, and the impact on short-term exposure to green spaces and mortality is still unknown. Besides mortality, green space has also been associated with morbidity outcomes, but there is little evidence from cohort studies on disease incidence or prevalence relating to green spaces. Our results should also be considered in the local social and political contexts. Although the benefits of green spaces and mortality that we found are robust, negative effects of increasing green spaces in the urban environment (such as gentrification) can occur, and these externalities should be considered when urban public policies are designed.

We found evidence of an inverse association between the proximity to green spaces and all-cause mortality. To our knowledge, this is the first meta-analysis of cohort studies in this area of research, providing robust evidence for policy recommendations. Our findings suggest that robust protocols should be used when studying the effects of green spaces on health, which should clearly define what is considered to be green space, how the study population was selected, and how data are collected, and that all relevant potential confounders should be accounted for. Additional studies of the associations between green spaces and mortality are needed, especially in low-income and middle-income countries, particularly of short-term mortality, morbidity, and in other populations, such as children. Finally, future policy interventions related to green spaces should consider the whole range of positive effects that are likely to affect the population, but they also need to be accompanied by regulations to reduce the possible negative effects of increased green space, such as crime and gentrification.
