
JOURNAL INFORMATION
==============================
NLM Title Abbreviation: Lancet Planet Health
Journal ID: Lancet Planet Health
NLM Journal ID: 101704339

The Lancet. Planetary Health

EISSN: 2542-5196

ARTICLE INFORMATION
==============================
PMCID: PMC8386287
PMID: 34390667
DOI: 10.1016/S2542-5196(21)00208-4
Article ID: S2542-5196(21)00208-4
Article version: 1
Subjects: Corrections

Correction to Lancet Planet Health 2019; 3: e469–77

Publication date: 2021 Aug
Electronic publication date: 2021 Aug 11
Volume: 5
Issue: 8
First page: e504
Last page: e504
Copyright: .
Copyright year: 2021
Copyright holder: Elsevier Ltd
License: This is an open access article under the CC BY license (http://creativecommons.org/licenses/by/3.0/).
License URL: https://creativecommons.org/licenses/by/3.0/
Erratum for: Green spaces and mortality: a systematic review and meta-analysis of cohort studies 3 11 11 2019 e469 e477 The Lancet. Planetary Health 10.1016/S2542-5196(19)30215-3 PMC6873641 31777338

==============================
Rojas-Rueda D, Nieuwenhuijsen MJ, Gascon M, Perez-Leon D, Mudu P. Green spaces and mortality: a systematic review and meta-analysis of cohort studies. Lancet Planet Health 2019; 3: e469–77—In figure 2 of this Article, the forest-plot boxes have been reordered to correctly correspond to the hazard ratios. This correction has been made as of Aug 11, 2021.

