# Supplementary material for: Green spaces and mortality: a systematic review and meta-analysis of cohort studies
Source: Lancet Planet Health. 2019 Nov;3(11):e469–77. doi: 10.1016/S2542-5196(19)30215-3 (PMC6873641; doi:10.1016/S2542-5196(19)30215-3)
Supplement: Supplementary appendix [file mmc1.pdf]

THE LANCET
Planetary Health

Supplementary appendix

This appendix formed part of the original submission and has been peer reviewed. We post it as supplied by the authors.

Supplement to: Rojas-Rueda D, Nieuwenhuijsen MJ, Gascon M, Perez-Leon D, Mudu P. Green spaces and mortality: a systematic review and meta-analysis of cohort studies. *Lancet Planet Health* 2019: **3:** e469–77.

1. Methods

1.1. PRISMA 2009 Checklist

Section/topic	#	Checklist item	Reported on page #
**TITLE**			
Title	1	Identify the report as a systematic review, meta-analysis, or both.	1
**ABSTRACT**			
Structured summary	2	Provide a structured summary including, as applicable: background; objectives; data sources; study eligibility criteria, participants, and interventions; study appraisal and synthesis methods; results; limitations; conclusions and implications of key findings; systematic review registration number.	2
**INTRODUCTION**			
Rationale	3	Describe the rationale for the review in the context of what is already known.	3
Objectives	4	Provide an explicit statement of questions being addressed with reference to participants, interventions, comparisons, outcomes, and study design (PICOS).	3
**METHODS**			
Protocol and registration	5	Indicate if a review protocol exists, if and where it can be accessed (e.g., Web address), and, if available, provide registration information including registration number.	5
Eligibility criteria	6	Specify study characteristics (e.g., PICOS, length of follow-up) and report characteristics (e.g., years considered, language, publication status) used as criteria for eligibility, giving rationale.	4
Information sources	7	Describe all information sources (e.g., databases with dates of coverage, contact with study authors to identify additional studies) in the search and date last searched.	4
Search	8	Present full electronic search strategy for at least one database, including any limits used, such that it could be repeated.	Supplemental material
Study selection	9	State the process for selecting studies (i.e., screening, eligibility, included in systematic review, and, if applicable, included in the meta-analysis).	4-5

Data collection process	10	Describe method of data extraction from reports (e.g., piloted forms, independently, in duplicate) and any processes for obtaining and confirming data from investigators.	4-5
Data items	11	List and define all variables for which data were sought (e.g., PICOS, funding sources) and any assumptions and simplifications made.	4-5
Risk of bias in individual studies	12	Describe methods used for assessing risk of bias of individual studies (including specification of whether this was done at the study or outcome level), and how this information is to be used in any data synthesis.	5 & supplemental material
Summary measures	13	State the principal summary measures (e.g., risk ratio, difference in means).	5
Synthesis of results	14	Describe the methods of handling data and combining results of studies, if done, including measures of consistency (e.g., I^2) for each meta-analysis.	5

Section/topic	#	Checklist item	Reported on page #
Risk of bias across studies	15	Specify any assessment of risk of bias that may affect the cumulative evidence (e.g., publication bias, selective reporting within studies).	5 & supplemental material
Additional analyses	16	Describe methods of additional analyses (e.g., sensitivity or subgroup analyses, meta-regression), if done, indicating which were pre-specified.	-
**RESULTS**			
Study selection	17	Give numbers of studies screened, assessed for eligibility, and included in the review, with reasons for exclusions at each stage, ideally with a flow diagram.	6
Study characteristics	18	For each study, present characteristics for which data were extracted (e.g., study size, PICOS, follow-up period) and provide the citations.	6 & table 1
Risk of bias within studies	19	Present data on risk of bias of each study and, if available, any outcome level assessment (see item 12).	Supplemental material
Results of individual studies	20	For all outcomes considered (benefits or harms), present, for each study: (a) simple summary data for each intervention group (b) effect estimates and confidence intervals, ideally with a forest plot.	table 1 and figure 2
Synthesis of results	21	Present results of each meta-analysis done, including confidence intervals and measures of	6 and figure 2

		consistency.	
Risk of bias across studies	22	Present results of any assessment of risk of bias across studies (see Item 15).	6
Additional analysis	23	Give results of additional analyses, if done (e.g., sensitivity or subgroup analyses, meta-regression [see Item 16]).	-
**DISCUSSION**			
Summary of evidence	24	Summarize the main findings including the strength of evidence for each main outcome; consider their relevance to key groups (e.g., healthcare providers, users, and policy makers).	6 and 7
Limitations	25	Discuss limitations at study and outcome level (e.g., risk of bias), and at review-level (e.g., incomplete retrieval of identified research, reporting bias).	8
Conclusions	26	Provide a general interpretation of the results in the context of other evidence, and implications for future research.	7, 8 and 9
**FUNDING**			
Funding	27	Describe sources of funding for the systematic review and other support (e.g., supply of data); role of funders for the systematic review.	5

From: Moher D, Liberati A, Tetzlaff J, Altman DG, The PRISMA Group (2009). Preferred Reporting Items for Systematic Reviews and Meta-Analyses: The PRISMA Statement. PLoS Med 6(7): e1000097. doi:10.1371/journal.pmed1000097

For more information, visit: **www.prisma-statement.org**.

1.2. Search strategy used in PUBMED (run on August 20th, 2019): 9298 results

((((Green space OR greenspace* OR greenness OR greenery OR wilderness OR wild land OR natural land OR natural environment OR municipal land OR community land OR public land OR open land OR wild space OR municipal space OR natural space OR open space OR municipal park OR park OR botanic park OR park access OR urban park OR city park OR park availability OR public garden OR natural neighbourhood OR natural facilities OR vegetation natural OR belt green OR wild area OR trail green OR natural area* OR green area* OR built environment OR urban design OR recreation resource OR woodland OR forest OR shinrin-yoku OR forest bathing OR NDVI OR Normalized Difference Vegetation Index))) AND ((“Mortality”[all fields] OR “Mortality”[MeSH] OR “all-cause mortality”[all fields]))) AND ((“Longitudinal studies”[all fields] OR “Longitudinal studies”[MeSH] OR “Cohort studies”[all fields] OR “Cohort studies”[MeSH]))

1.3. Risk of bias

We evaluated the risk of bias by means of a checklist developed by the WHO (2012) and Van Kempen (2018): (i) information bias due to exposure assessment, (ii) bias due to confounding, (iii) bias due to selection of participants, (iv) information bias I due to health outcome assessment, and (v) information bias II due to health outcome assessment. For each study, the evaluation was carried out by two independent reviewers (DRR and DPL). Table 1 shows how we scored the studies on these items. From these scores, we calculated a total risk of bias score. For studies where there was a difference of opinion between the two reviewers, we attempted to reach consensus through discussion between them.

Table 1. Risk of bias assessment (WHO 2012*and van Kempen et al., 2018**).

	**Bias due to exposure assessment**	**Bias due to confounding**	**Bias due to selection of participants**	**Bias due to health outcome assessment**	**Bias due to not blinded outcome assessment**	**Total risk of bias**
Low	A clear description of the exposure assessment and exposure unit; based on measurements or modeling.	All important confounders are taken into account either through matching or, restriction or in the analysis. (e.g., age, gender, etc.)	Participants randomly sampled from a known population, AND response rate higher than 60%, AND attrition rate less than 20% in follow-up studies.	The health outcome of interest is objectively measured OR taken from medical records OR taken from questionnaire or interview using a known scale or validated assessment method.	The health outcome of interest is assessed blind for exposure information in cohort and cross-sectional studies or exposure is assessed blind for being a case in case-control studies	At least 4 at low risk of bias. One “high” or “unclear” out of five is allowed.
High	Not clear description of the exposure assessment or exposure unit OR/AND performed by unqualified staff	Only 1 or no confounder is taken into account; OR subjects in exposed and unexposed groups differ for one or more important confounders and there is no adjustment in the analysis	No random sampling OR response rate less than 60% OR attrition rate higher than 20%.	The health outcome of interest is self-reported and not assessed using a known scale or validated assessment method	The health outcome and/or exposure assessment is not blinded.	Any other.
Unclear	If not enough information is available to judge the above	Less then all to > 1 important confounders taken into account, OR Insufficient information to decide on one of the above.	No information to judge the above.	Not sufficient information reported to assess the above.	Not sufficient information reported to assess the above.	
Not Apply		NA	NA		NA	

*World Health Organization, WHO Handbook for guideline development. 2012, Geneva: World Health Organization.

van Kempen, E.; Casas, M.; Pershagen, G.; Foraster, M. WHO Environmental Noise Guidelines for the European Region: A Systematic Review on Environmental Noise and Cardiovascular and Metabolic Effects: A Summary. *Int. J. Environ. Res. Public Health* **2018, *15*, 379.

2.Results

Table 2. Risk of bias assessment from each study included in the meta-analysis.

Study	Reason for bias					
	Exposure assessment	Confounding	Selection of participants	Health outcome assessment	Not blinded outcome assessment	Total risk of bias
**Crouse et al. (2017), Canada**	Low	Low	Low	Low	High	Low
**James et al. (2016), USA**	Low	Low	High	Low	High	High
**Ji et al. (2019), China**	Low	Low	Low	Low	High	Low
**Nieuwenhuijsen et al. (2018), Spain**	Low	High	Low	Low	High	High
**Orioli et al. (2019), Italy**	Low	Low	Low	Low	High	Low
**Vienneau et al. (2017), Switzerland**	Low	High	Low	Low	High	High
**Villeneuve et al. (2012), Canada**	Low	Low	Low	Low	High	Low
**Wilker et al. (2014), USA**	Low	Low	Low	Low	High	Low
**Zijlema et al. (2019), Australia**	Low	Low	Low	Low	High	Low

Figure 1. Funnel plot (publication bias), of the association between greenness and all-cause mortality for each 0.1 increment of NDVI.

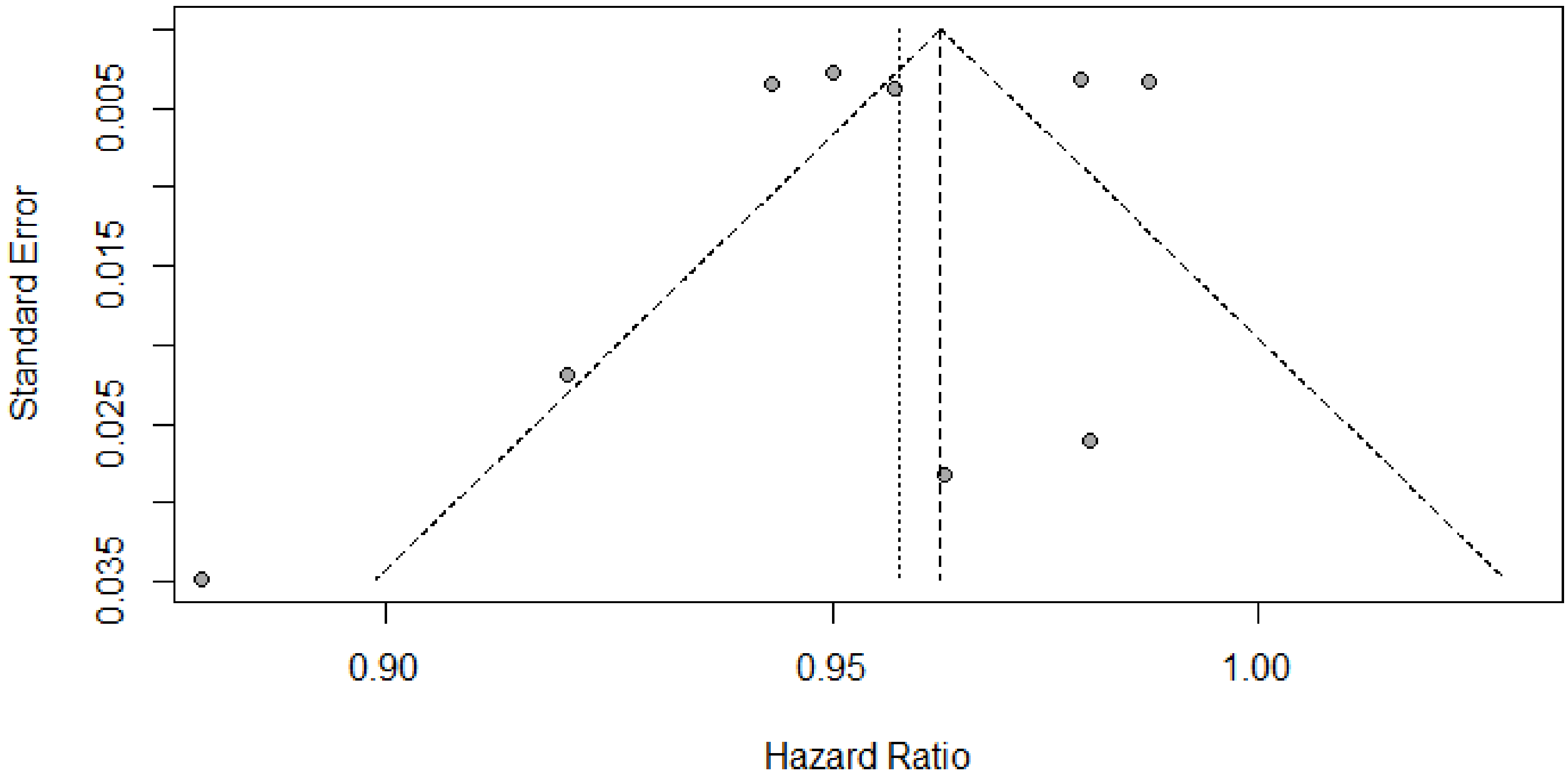

Figure 2. Trim-and-fill funnel plot (publication bias), of the association between greenness and all-cause mortality for each 0.1 increment of NDVI. Darker dots represent the eight studies included in the meta-analysis. Lighter dots represent the two studies added by trim and fill methods to reduce asymmetry.

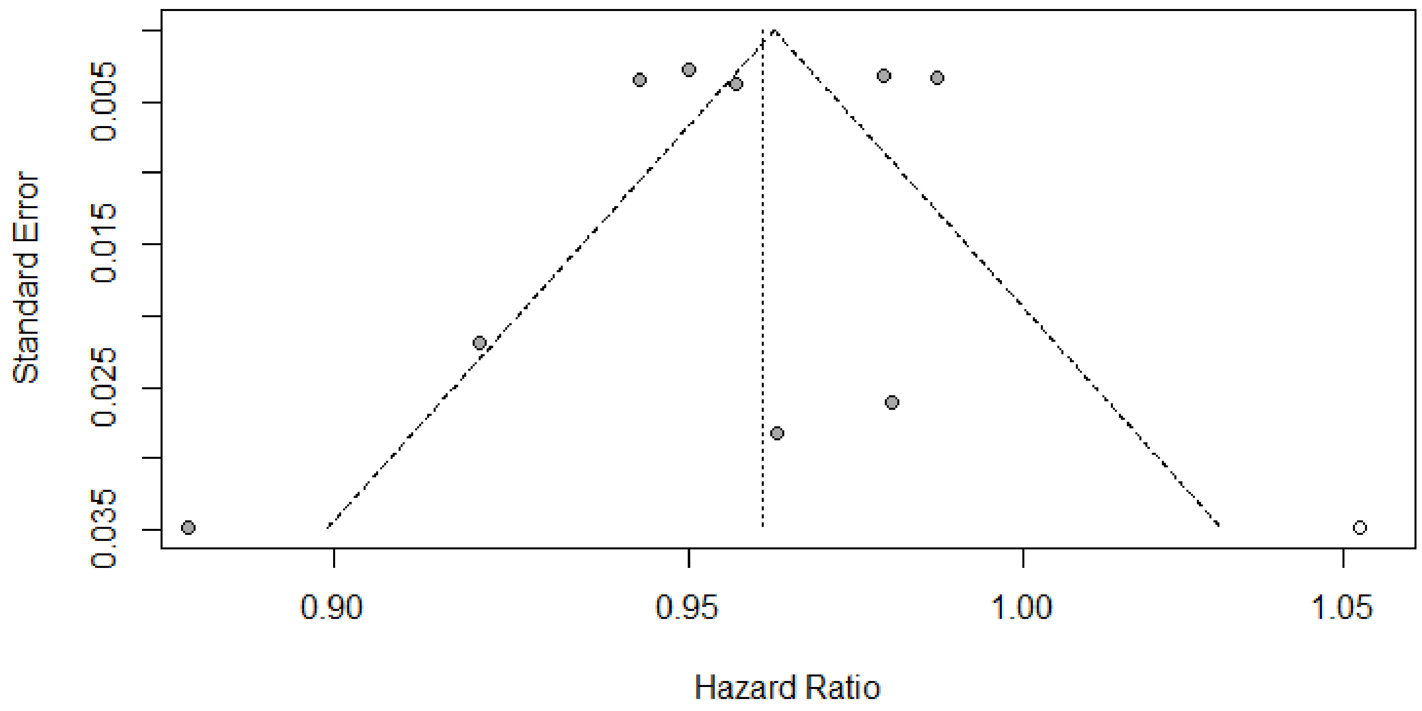

Trim-and-fill funnel plot (publication bias), of the association between greenness and all-cause mortality for each 0.1 increment of NDVI.

```
                  HR            95%-CI %W(random)
Crouse         0.9430 [0.9365; 0.9495]       14.9
James          0.8800 [0.8219; 0.9422]        3.8
Ji             0.9500 [0.9450; 0.9550]       15.1
Nieuwenhuijsen 0.9200 [0.8812; 0.9605]        6.9
Orioli         0.9870 [0.9805; 0.9935]       14.9
Vienneau       0.9570 [0.9500; 0.9640]       14.8
Villeneuve     0.9790 [0.9730; 0.9850]       15.0
Wilker         0.9630 [0.9111; 1.0179]        5.1
Zijlema        0.9800 [0.9312; 1.0313]        5.7
Filled: James  1.0529 [0.9834; 1.1274]        3.8

Number of studies combined: k = 10 (with 1 added studies)

                         HR            95%-CI     z  p-value
Random effects model 0.9609 [0.9463; 0.9758] -5.10 < 0.0001

Quantifying heterogeneity:
tau^2 = 0.0004; H = 4.26 [3.47; 5.22]; I^2 = 94.5% [91.7%; 96.3%]

Test of heterogeneity:
      Q d.f.  p-value
 163.30    9 < 0.0001
```
